# GALNT14 genotype as a response predictor for concurrent chemoradiotherapy in advanced esophageal squamous cell carcinoma

**DOI:** 10.18632/oncotarget.16253

**Published:** 2017-03-16

**Authors:** Yung-Kuan Tsou, Kung-Hao Liang, Wey-Ran Lin, Hsien-Kun Chang, Chen-Kan Tseng, Chau-Ting Yeh

**Affiliations:** ^1^ Department of Hepato-Gastroenterology, Chang Gung Memorial Hospital, Taoyuan, Taiwan; ^2^ Liver Research Center, Chang Gung Memorial Hospital, Taoyuan, Taiwan; ^3^ Molecular Medicine Research Center, Chang Gung University, Taoyuan, Taiwan; ^4^ Chang Gung University, College of Medicine, Taoyuan, Taiwan; ^5^ Department of Oncology, Chang Gung Memorial Hospital, Taoyuan, Taiwan; ^6^ Department of Radiation Oncology, Chang Gung Memorial Hospital, Taoyuan, Taiwan

**Keywords:** germline polymorphism, biomarker, cisplatin, gastrointestinal cancer, pharmacogenomics

## Abstract

Esophageal squamous cell carcinoma is an aggressive cancer. We investigated genetic response predictors for patients with advanced esophageal squamous cell carcinoma receiving concurrent chemoradiotherapy. A cohort of 108 patients was recruited. Survival analysis showed that lower esophageal location of tumor, more advanced metastasis stage, and longer length of tumor were associated with poorer overall survival (adjusted P = 0.001, < 0.001, and 0.045, respectively), while the presence of complete/partial response to concurrent chemoradiotherapy was independently associated with better overall survival (adjusted P < 0.001). The *GALNT14*-rs9679162 “GG” genotype was associated with a lower rate of response (P = 0.014). Multivariate Cox-proportional hazards models also showed that the “GG” genotype was associated with a longer time to complete/partial response (adjusted P = 0.022), independent of leukocyte counts and gender. In conclusion, the presence of a complete/partial response to chemoradiotherapy was critical for advanced esophageal squamous cell carcinoma patients to achieve better overall survival. The *GALNT14*-rs9679162 “GG” genotype was associated with a longer time to complete/partial response of concurrent chemoradiotherapy.

## INTRODUCTION

Esophageal cancer is the 8^th^ most prevalent cancer around the globe [1]. Esophageal squamous cell carcinoma (ESCC) and adenocarcinoma are two major types of esophageal cancer. The global age-standardized incidence rate of ESCC is 1.4-13.6 per 100,000 people [1]. Patients with early, localized esophageal cancer can benefit from esophagectomy [2]. Patients with locally advanced, unresectable esophageal cancer are treated with various chemotherapy regimens or concurrent chemoradiotherapy (CCRT) to prolong their survival and/or to improve their quality of life [3–6]. A randomized trial have demonstrated the superiority of CCRT in comparison with radiotherapy alone [7]. Besides, CCRT has been shown an effective therapeutic modality for neoadjuvant treatment before surgical resections [6, 8, 9]. The most frequently used chemotherapy regimens are a combination of 5-fluorouracil and cisplatin [10]. Clinically, a wide diversity of response rates of CCRT has been observed (15 to 45%) [10]. Unfortunately, ~50% of the esophageal cancer patients, when first diagnosed, were already at a far advanced stage with cancer metastasis [11]. Patients of this stage have a mean survival time less than 8.1 months [10]. The standard treatments have not been established for metastatic esophageal cancer [2]. It remained elusive whether the chemoradiotherapeutic response can be translated to survival benefits in advanced esophageal cancer patients [2, 6].

CCRT has a high-toxicity profile which restricts its clinical use. The capability to predict therapeutic responses are thus urgently needed for clinicians to select suitable patient groups for aggressive treatments [12, 13]. Many potential therapeutic response predictors have been reported for early or locally advanced esophageal cancer in the literature [14–22]. However, to date, no genetic response predictor has ever been identified for chemoradiotherapy in ESCC patients with cancer metastasis. Recently, the genotypes of the *GALNT14*-rs9679162 single nucleotide polymorphism (SNP) has been shown to be an effective predictor for systemic chemotherapy response in advanced hepatocellular carcinoma (HCC), based on a genome-wide exploration of 500,000 SNPs in human white blood cells [23], and subsequent prospective [24] and retrospective validations [25, 26]. This genotype was further demonstrated to be an effective outcome predictor of transcatheter arterial chemoembolization treatment (TACE) for intermediate-stage HCC patients [27]. In all these studies, the *GALNT14* rs9679162 “T” allele was associated to favorable outcomes of patients, while the “G” allele was associated to poor outcomes. The systemic combination chemotherapy regimens used for the previous studies were 5- fluorouracil, cisplatin and mitoxantrone, of which two were commonly used for advanced ESCC. Furthermore, the GALNT14 protein has been shown to be able to enhance the extrinsic apoptotic signaling of cancer cells [27, 28]. It would be interesting to know if *GALNT14* genotype could be a predictor of chemoradiotherapeutic response in patients with advanced ESCC.

Therefore, the main goals of this study were (i) to examine whether the chemoradiotherapeutic response was associated with survival benefits in advanced ESCC patients; and (ii) whether the *GALNT14* genotypes were associated with the chemoradiotherapeutic response.

## RESULTS

### Baseline characteristics of the patients

A total of 108 patients were included, and their basic clinical data were listed in Table 1. Most patients were male (96.3%). Most of them had an ECOG performance status score of 1 or 0 (83.3%). Among all patients, 34.3% had tumors across two regions. The percentages of patients with tumors located only in the upper, middle, or lower esophagus were 19.4%, 25.0% and 21.3%, respectively. The most common histological grading was moderately differentiated (67.9%), followed by poorly differentiated (25.5%). Tumor length was 7.6 ± 3.7 cm. With regard to tumor stage, 45.4% of the patients had T3 and 46.3% had T4 diseases. When considering the metastasis stage, 38%, 12%, and 50%, respectively, had regional LNs, distant LNs, and organ metastasis.

**Table 1:** Baseline clinical data of the 108 advanced esophageal cancer patients included

Parameters	
Age, years, mean ± SD	52.6 ± 9.4
Gender, Male (%)	104 (96.3)
Location of cancer	
Upper esophagus (%)	21 (19.4)
Middle esophagus (%)	27 (25.0)
Lower esophagus (%)	23 (21.3)
Cross two regions (%)	37 (34.3)
Histology grading	
Well differentiated (%)	4 (3.7)
Moderate differentiated (%)	72 (67.9)
Poorly differentiated (%)	27 (25.5)
Not graded (%)^a^	3 (2.8)
Tumor stage	
T1/ T2/ T3/ T4 (%)	1/ 8/ 49/ 50 (0.9/ 7.4/ 45.4/ 46.3)
Metastasis stage	
Regional N/ Distant N/ O (%)^b^	41/ 13/ 54 (38.0/ 12.0/ 50.0)
ECOG stage	
Stage 0/ 1	16/ 74 (14.8/ 68.5)
> 1	18 (16.7)
Tumor length, cm, mean ± SD	7.6 ± 3.7
Biochemistry and hemogram	
Albumin, g/dL, mean ± SD	3.7 ± 0.6
Alanine transaminase, U/L, mean ± SD	21.4 ± 15.6
Creatinine, mg/dL, mean ± SD	0.7 ± 0.3
Bilirubin, mg/dL, mean ± SD	0.6 ± 0.5
Leukocytes, × 10^9^/L, mean ± SD	9.3 ± 4.2
Neutrophil percentages, %, mean ± SD	70.4 ± 11.6
Hemoglobin, g/dL, mean ± SD	12.0 ± 2.2
*GALNT14* genotype	
TT	28 (25.9)
GG	29 (26.9)
TG	51 (47.2)

^a^Histology was accessed at other hospitals.

^b^N, lymph node; O, organ.

In this cohort, the numbers of patients of the *GALNT14* rs9679162 “TT”, “TG” and “GG” genotypes were 28 (25.9%), 51 (47.2%) and 29 (26.9%), respectively. This genotype distribution did not deviate significantly from those of the HapMap Chinese Han Beijing (CHB) and Metropolitan Denver (CHD) ethnic reference cohorts (Cochran-Armitage Trend test, P = 0.422 and 0.575, respectively).

### Complete/partial responses to CCRT was positively associated with overall survival, independent of tumor locations, metastasis stages and tumor lengths

Therapeutic responses of CCRT were analyzed for their association with overall survival, alongside other clinical variables. Based on the RECIST definition [29], patients were classified into two groups: the responder group which included patients with complete and partial responses, respectively; and the non-responder group which included patients with stable disease and progressive disease, respectively. In the univariate analysis, tumor location, metastasis stage, ECOG status, tumor length, pre-treatment serum levels of albumin and alanine transaminase, level of hemoglobin, and therapeutic response (including complete and partial responses) to CCRT were associated significantly with the overall survival (Table 2).

**Table 2:** Cox proportional hazard analysis for overall survival in relation to clinical parameters

Clinical parameters	Univariate analysis			Multivariate analysis		
	HR	95% CI	P	HR	95% CI	P
Age, per year increase	0.991	0.973 – 1.010	0.359			
Gender, Male = 1	0.469	0.171 – 1.286	0.141			
Location of tumor						
Upper = 1	0.712	0.440 – 1.151	0.165			
Middle = 1	1.076	0.691 – 1.675	0.746			
Lower = 1	1.759	1.090 – 2.839	**0.021**	2.462	1.416 – 4.281	**0.001**
Histology, Poorly differentiated = 1	1.198	0.778 – 1.846	0.412			
Tumor stage, per stage increase	1.235	0.912 – 1.673	0.173			
Metastasis stages, per stage increase^a^	1.434	1.159 – 1.775	**0.001**	1.659	1.253 – 2.196	**< 0.001**
ECOG Stage, greater than one = 1	2.062	1.230 – 3.458	**0.006**	0.661	0.357 – 1.225	0.188
Tumor length, per mm increase	1.009	1.003 – 1.014	**0.002**	1.007	1.000 – 1.013	**0.045**
Albumin, per g/dL increase	0.580	0.388 – 0.868	**0.008**	0.832	0.529 – 1.307	0.424
Alanine transaminase, per U/L increase	1.020	1.008 – 1.033	**0.001**	1.008	0.995 – 1.022	0.213
Creatinine, per mg/dL increase	1.013	0.456 – 2.251	0.974			
Bilirubin, per mg/dL increase	1.142	0.771 – 1.691	0.509			
Leukocytes, per × 10^9^/L increase	1.050	0.999 – 1.103	0.054			
Neutrophil percentage, per % increase	2.676	0.505 – 14.187	0.247			
Hemoglobin, per g/dL increase	0.890	0.814 – 0.973	**0.010**	0.947	0.851 – 1.053	0.311
Complete/partial response = 1	0.474	0.320 – 0.702	**< 0.001**	0.360	0.227 – 0.572	**< 0.001**

^a^Stage 1, 2, and 3 were defined as regional lymph nodes involvement only, distant lymph nodes involvement, and distant organ metastasis.

In the multivariate analysis, tumor location, metastasis stage, tumor length, and complete/partial response to CCRT remained significantly associated to overall survival. Patients with tumor in lower esophagus demonstrated poorer overall survival than in other locations (adjusted hazard ratio = 2.462, P = 0.001). Significant higher hazards were associated with an increment of metastasis stages (adjusted hazard ratio = 1.659, P < 0.001), where the stage 1, 2, and 3 indicated respectively regional lymph nodes involvement only, distant lymph nodes involvement, and distant organ metastasis. A longer length of the largest tumor was also associated significantly with poorer overall survival (adjusted hazard ratio = 1.007, P = 0.045).

The adjusted hazard ratio of complete/partial responses to overall survival was 0.360 (95% confidence interval = 0.227 – 0.572). As such, the presence of complete/partial responses to CCRT was critical for the patients to achieve a longer overall survival, independent of tumor location, metastasis stages, tumor size and other characteristics.

### GALNT14 genotype “GG” was significantly associated with poorer therapeutic responses of CCRT

*GALNT14* rs9679162 genotype distributions were significantly associated with therapeutic responses by the Cochran-Armitage Trend test (P = 0.047). We also analyzed dichotomized patient strata using genotypes: (1) “GG” versus “TT+TG”; and (2) “TT” versus “GG+TG”, to accommodate both the dominant and receive modes of inheritance. In (1), a significant association was found (P = 0.014, Table 3). 24.1% of the GG-typed patients had complete or partial response, in contrast to 50.6% of the TT/TG-typed patients. In (2), no significant difference was found (P = 0.422). Therefore, we used the dichotomized strata of patients with genotype “GG” and “TT+TG” respectively for all the following analysis.

**Table 3:** The association between the therapeutic responses of CCRT and *GALNT14* genotypes

*GALNT14* genotype	Therapeutic responses			P
	Number of patients	SD + PD	CR + PR	
“GG”	29	22 (75.9%)	7 (24.1%)	
“TT+TG”	79	39 (49.4%)	40 (50.6%)	**0.014**
“TT”	28	14 (50.0%)	14 (50.0%)	
“GG+TG”	80	47 (58.8%)	33 (41.3%)	0.422

SD, stable disease; PD, progressive disease.

CR, complete response; PR, partial response.

### GALNT14 “GG” was associated with longer time-to-complete/partial response, independent of gender and leukocyte counts

**Figure 1:**
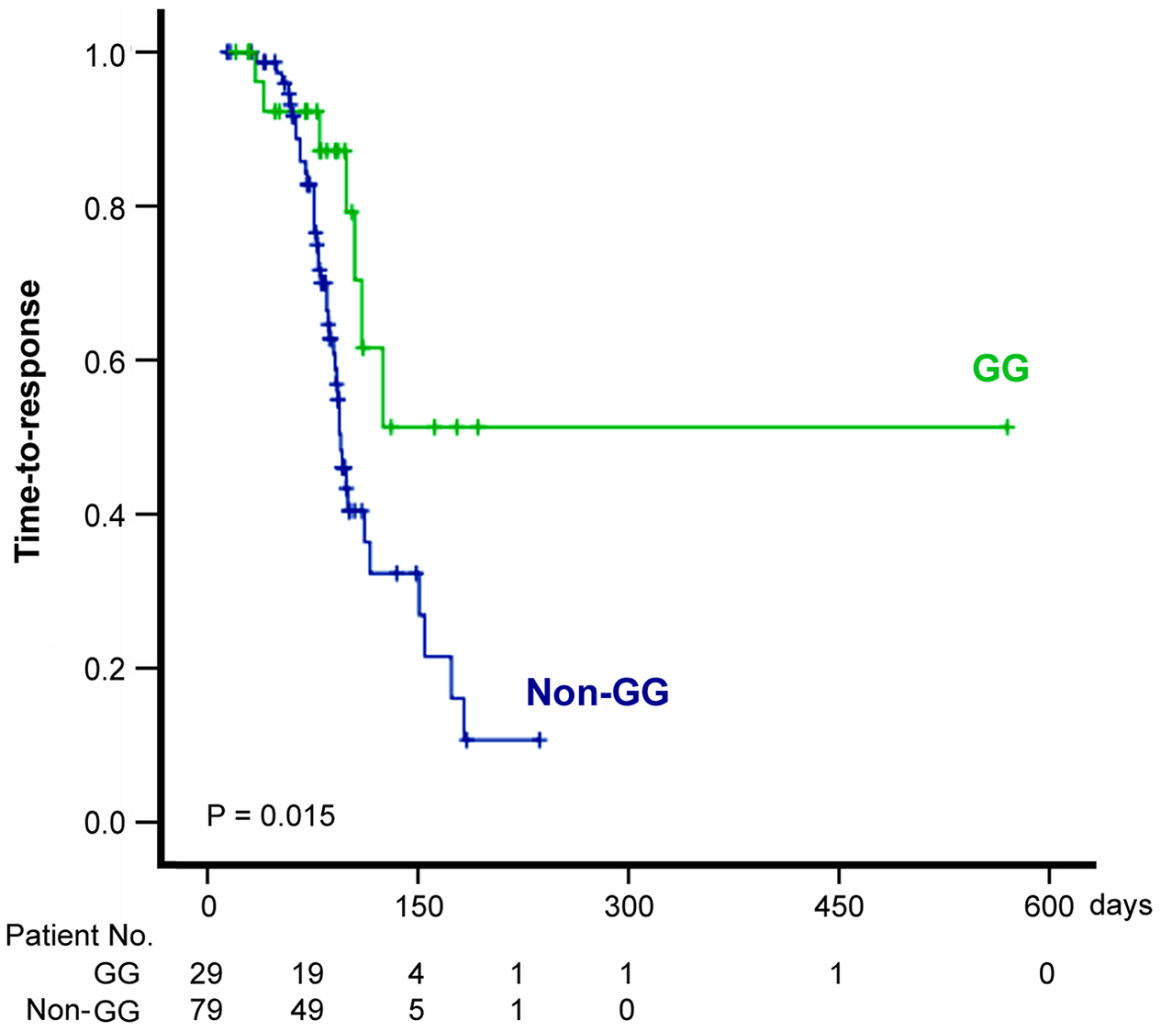
Kaplan-Meier curve of the time to response (including complete and partial response) of patients stratified by the *GALNT14* “GG” and “non-GG” genotypes Patients with the “GG” genotype showed longer time to response (log-Rank P = 0.015) than those with the “non-GG” genotype.

We further conducted a univariate/multivariate analysis on the time-to-responses to CCRT using the Cox proportional hazards model. Since the clinical events analyzed here (complete or partial responses) were favorable events rather than hazardous events, a hazard ratio larger than 1 actually indicated favorable predictors (shorter time-to-responses). Univariate analysis revealed that pre-treatment leukocyte count, gender and the *GALNT14* genotype were associated to time to complete/partial responses (Table 4). In the multivariate analysis of the three variables, only the *GALNT14* genotypes and the pre-treatment leukocyte count remained statistically significant. Patients with the *GALNT14* genotype “GG” showed longer time to complete/partial responses than those with the “TT” or “TG” genotype (adjusted hazard ratio = 0.385, P = 0.022). This was also demonstrated in the Kaplan-Meier time-to-response curves (Figure 1) where the genotype “GG” was associated with poor outcomes (log Rank P = 0.015). Also, patients with higher pre-treatment leukocyte counts showed shorter time to complete/partial responses (adjusted hazard ratio = 1.087, P = 0.014). The significant result in the multivariate analysis showed that *GALNT14* genotypes and the pre-treatment leukocyte count were independently associated with the time to complete/partial response of CCRT.

**Table 4:** Cox proportional hazard analysis for time-to-CCRT responses (including complete and partial response) in relation to clinical parameters

Clinical parameters	Univariate analysis			Multivariate analysis		
	HR	95% CI	P	HR	95%CI	P
Age, per year increase	1.008	0.980 – 1.036	0.595			
Gender, Male vs. Female	0.203	0.047 – 0.886	**0.034**	0.242	0.055 – 1.064	0.060
Location of tumor						
Upper vs. other	0.939	0.453 – 1.950	0.867			
Middle vs. other	1.130	0.604 – 2.116	0.703			
Lower vs. other	1.539	0.726 – 3.262	0.261			
Histology, Poorly differentiated = 1	0.927	0.479 – 1.794	0.822			
Tumor stage, per stage increase	1.222	0.788 – 1.896	0.371			
Metastasis stage, per stage increase	1.080	0.793 – 1.472	0.623			
ECOG Stage, greater than one = 1	0.476	0.166 – 1.367	0.168			
Tumor length, per cm increase	1.007	1.000 – 1.015	0.065			
Albumin, per g/dL increase	1.605	0.895 – 2.877	0.112			
Alanine transaminase, per U/L increase	1.004	0.983 – 1.025	0.721			
Creatinine, per mg/dL increase	0.716	0.264 – 1.943	0.511			
Bilirubin, per mg/dL increase	0.902	0.424 – 1.920	0.790			
Leukocytes, per × 10^9^/L increase	1.084	1.016 – 1.156	**0.014**	1.087	1.017 – 1.161	**0.014**
Neutrophil percentage, per % increase	5.022	0.439 – 57.443	0.194			
Hemoglobin, per g/dL increase	1.109	0.951 – 1.292	0.186			
*GALNT14* genotype, “TT” vs. “TG+GG”	1.423	0.759 – 2.671	0.272			
*GALNT14* genotype, “GG” vs. “TT+TG”	0.381	0.170 – 0.855	**0.019**	0.385	0.171 – 0.869	**0.022**

Distribution of *GALNT14* rs9679162 genotypes with respect to gender, location of tumor and metastasis stages were shown in Supplementary Table 1. To further evaluate any potential confounding relationships between *GALNT14* genotypes and all the other baseline variables, a logistic regression analysis was performed. The result showed that the *GALNT14* genotype (“GG” versus “TT+TG”) did not associate significantly with any other variable, including gender and pretreatment leucocyte counts (Supplementary Table 2).

## DISCUSSION

In our hospital, CCRT was frequently given as a palliative treatment in ESCC patients with cancer metastasis to relieve esophageal obstruction. The present study demonstrated that ~43.52% of such patients can achieve complete/partial response by CCRT. The complete/partial response was positively associated with better overall survival (Adjusted P < 0.001), independent of tumor location, length and metastasis stage. Therefore, a complete/partial response to CCRT should be rigorously pursued in the initial stage of treatment, as they can be translated into longer overall survival.

The next question was that whether the CCRT responses can be predicted effectively before treatment. The current study demonstrated that the *GALNT14* genotyping can serve as a tool for clinicians to decide in the commencing stage of CCRT whether CCRT alone is sufficient to achieve a complete or partial response. The *GALNT14* genotype, “TT+TG”, was associated with favorable treatment responses to CCRT in advanced ESCC patients, while the genotype “GG” was associated with unfavorable outcomes (Table 3). This result was in agreement with the chemotherapy or chemoembolization treatment of unresectable hepatocellular carcinoma, where the “T” allele consistently indicated favorable outcomes [23, 24, 27]. The percentages of “TT+TG” patients in this study cohort, as well as in the HapMap Chinese Han Beijing (CHB) and Metropolitan Denver (CHD) ethnic reference cohorts, were 73.2%, 76.5% and 76.2% respectively. This implied that a majority of ESCC patients with cancer metastasis were potential responders to CCRT, while those with the “GG” genotype may need other novel treatments, for example, the addition of docetaxel to CCRT [3, 10, 30].

The *GALNT14* gene encodes a glycosyltransferase GalNac-T14 which is involved in protein post-translational O-linked glycosylation. It catalyzes the initiation of glycosylation, by conjugating the sugar molecule N-acetyl-D-galactosamine (GalNac) into the Serine or Threonine residues of the protein. On top of the GalNac moiety, further glycosylation can take place. In a genome-wide investigation of consanguineous families, *GALNT14* was identified to bear damaging Mendelian mutations which caused embryonic lethality, suggesting the irreplaceable role of GalNac-T14 in human development [31]. Germline mutations were also found in a recent study on the congenital disorders of glycosylation [32]. Finally, a genomic-screening of familial neuroblastoma also identified germline mutations which were responsible for the cancer [33]. Our recent studies also indicated a tight association between *GALNT14* genotype and chemotherapy or chemoembolization responses in advanced hepatocellular carcinoma patients. It is therefore not surprising to find that *GALNT14* genotype also associated with CCRT responses in advanced ESCC patients.

Recently, a single nucleotide polymorphism (rs9331888) in the 5′ untranslated region of the Clusterin gene was shown to be associated with the occurrence of ESCC based on a cross-sectional comparison between ESCC patients and healthy control subjects [34]. The “GG” genotype was shown to associate with higher risk of ESCC than the “CC+CG” genotypes [34]. In terms of treatments by definitive chemoradiotherapy, one other study showed that low Clusterin levels, detected by immunohistochemistry, were associated with complete response [17]. Despite these evidence, the roles of Clusterin in patients with distant metastasis remained unclear because such patients were excluded from previous studies. The higher expression levels of vascular endothelial growth factor (VEGF), measured by immunohistochemistry, was associated with higher rate of complete response of definitive CCRT in a multivariate analysis [21]. However, a meta-analysis of 19 studies on a wide spectrum of ESCC patients showed that VEGF positivity actually correlated with poor prognosis of ESCC patients [35]. Thus, conflicting evidence of VEGF in ESCC remained to be resolved.

In conclusion, a complete/partial response to CCRT in advanced ESCC patients is critical for them to achieve a longer survival. The *GALNT14* genotype “TT+TG” were associated with a higher chance of complete/partial response to CCRT, whereas the “GG” genotype was associated with an unfavorable treatment outcome. Other adjuvant treatment, such as chemotherapies or targeted therapies may be needed for patients with the “GG” genotype.

## MATERIALS AND METHODS

### Patients

This retrospective study was approved by the institutional review board of Chang Gung Memorial Hospital (ID:103-3422B), conducted under the provisions of the Declaration of Helsinki. A computer database in cancer register center, Chang Gung Memorial Hospital, Linkou medical center was searched for patients satisfying the following inclusion criteria: patients who had newly diagnosed clinical stage IV ESCC based on endoscopic biopsy and imaging study from January 2007 to May 2013; patients received CCRT with chemotherapy regimen of a combination of cisplatin plus 5-fluorouracil; patients who were followed regularly; and patients who died of ESCC. A total of 135 patients fulfilled the inclusion criteria. Among them, 27 patients did not have specimens available in the Tissue bank of Chang Gung Memorial Hospital, and were excluded from this study. As a result, 108 patients were analyzed in this study. All of them have given informed consent before depositing their samples to the Tissue Bank. The CCRT protocol has been published previously [8, 9]. Briefly, cisplatin was given by intravenous infusion, 75 mg/m^2^ per day over 3 hours on day 1, and 5-fluorouracil was given 1000 mg/m^2^ per day, by continuous infusion over 96 hours at days 1-4, repeated every 28 days, 2-4 cycles. Radiation was given between days 8 and 29 for 200 cGy per daily fraction, 5 days a week, to a total dose of 30 Gy. The treatment efficacy was evaluated by Computed Tomography (CT), taken before and 4-6 weeks after the treatment, according to the Response Evaluation Criteria in Solid Tumours (RECIST) definition [29].

The patients were given CCRT because they were diagnosed as stage IV ESCC patients under the definitions of the American Joint Committee on Cancer (AJCC) 6^th^ edition before 2010 and 7^th^ edition [36] after 2010. Since the definitions of the two editions was slightly different, we re-scored the metastatic status of all patients according to the definition of AJCC 7^th^ edition as: (1) regional lymph node (LN) metastasis, defined as any periesophageal LN from cervical nodes to celiac nodes; (2) distant LN metastasis, defined as LN metastasis beyond the regional LNs without organ metastasis; and (3) organ metastasis. Clinical parameters were collected and recorded, including age, gender, tumor locations and stages, histologic grade, Eastern Cooperative Oncology Group (ECOG) performance status [37], and tumor length (based on CT scans). Biochemistry and hemogram analysis included albumin, bilirubin, alanine transaminase (ALT), creatinine, leukocyte count, percentage of neutrophils, and hemoglobin.

### GALNT14 genotyping

G*e*notyping of *GALNT14* was performed using the previously-described technology [24, 25]. In short, nuclear DNA was extracted and purified from the non-tumor part of the formalin-fixed, paraffin-embedded esophageal biopsied specimens. The primers were as follows: forward, 5′-TCACGAGGCCAACATTCTAG-3′ and reverse, 5′-TTAGATTCTGCATGGCTCAC-3′. They were used for PCR and direct sequencing for a 172-bp intronic region of *GALNT14* covering rs9679162. The SNPs determined from both directions of sequencing were completely matched in all samples.

### Statistical analysis

Genotype data were analyzed by Cochran-Armitage Trend test. Dichotomized data were presented as ratios (%) and compared by the Chi-square tests. Survival analysis was performed by analyzing time to events from the date of initial treatment to the occurrence of events or loss of follow-up, where the events included death and therapeutic response defined by the RECIST criteria [29]. The loss of follow up was treated as the censored data in the survival analysis. The univariate and multivariate Cox proportional hazards model was used for the survival analyses with respect to clinical and genotypic variables. Multivariable analysis was performed only on variables which showed significant associations in the univariate analysis. Following dichotomized stratification of patients by their genotypes, the Kaplan-Meier method was used to estimate the survival probability between groups, and the log-rank test was used to compare survival outcomes. P<0.05 was considered a statistically significant difference. The SPSS version 17 software were used for the analysis (SPSS, Inc., Chicago, IL, USA).

## SUPPLEMENTARY MATERIALS TABLES



## References

[1] Vizcaino AP, Moreno V, Lambert R, Parkin DM (2002). Time trends incidence of both major histologic types of esophageal carcinomas in selected countries, 1973-1995. International Journal of Cancer.

[2] Varghese TK, Hofstetter WL, Rizk NP, Low DE, Darling GE, Watson TJ, Mitchell JD, Krasna MJ (2013). The Society of Thoracic Surgeons Guidelines on the Diagnosis and Staging of Patients With Esophageal Cancer. The Annals of Thoracic Surgery.

[3] Higuchi K, Koizumi W, Tanabe S, Sasaki T, Katada C, Azuma M, Nakatani K, Ishido K, Naruke A, Ryu T (2009). Current management of esophageal squamous-cell carcinoma in Japan and other countries. Gastrointest Cancer Res.

[4] Crosby TD, Brewster AE, Borley A, Perschky L, Kehagioglou P, Court J, Maughan TS (2004). Definitive chemoradiation in patients with inoperable oesophageal carcinoma. Br J Cancer.

[5] Honing J, Smit JK, Muijs CT, Burgerhof JG, de Groot JW, Paardekooper G, Muller K, Woutersen D, Legdeur MJ, Fiets WE, Slot A, Beukema JC, Plukker JT (2014). A comparison of carboplatin and paclitaxel with cisplatinum and 5-fluorouracil in definitive chemoradiation in esophageal cancer patients. Annals of Oncology.

[6] Stahl M, Budach W, Meyer HJ, Cervantes A (2010). Esophageal cancer: Clinical Practice Guidelines for diagnosis, treatment and follow-up. Annals of Oncology.

[7] Cooper JS, Guo MD, Herskovic A, Macdonald JS, Martenson JA, Al-Sarraf M, Byhardt R, Russell AH, Beitler JJ, Spencer S, Asbell SO, Graham MV, Leichman LL (1999). Chemoradiotherapy of Locally Advanced Esophageal Cancer. JAMA.

[8] Chao YK, Tseng CK, Wen YW, Liu YH, Wan YL, Chiu CT, Chang WC, Chang HK (2013). Using Pretreatment Tumor Depth and Length to Select Esophageal Squamous Cell Carcinoma Patients for Nonoperative Treatment After Neoadjuvant Chemoradiotherapy. Annals of Surgical Oncology.

[9] Huang RW, Chao YK, Wen YW, Chang HK, Tseng CK, Chan SC, Liu YH (2014). Predictors of pathological complete response to neoadjuvant chemoradiotherapy for esophageal squamous cell carcinoma. World J Surg Onc.

[10] Tanaka Y, Yoshida K, Yamada A, Tanahashi T, Okumura N, Matsuhashi N, Yamaguchi K, Miyazaki T (2016). Phase II trial of biweekly docetaxel, cisplatin, and 5-fluorouracil chemotherapy for advanced esophageal squamous cell carcinoma. Cancer Chemotherapy and Pharmacology.

[11] Ilson DH (2003). Oesophageal cancer: new developments in systemic therapy. Cancer Treatment Reviews.

[12] Chen M, Huang J, Zhu Z, Zhang J, Li K (2013). Systematic review and meta-analysis of tumor biomarkers in predicting prognosis in esophageal cancer. BMC Cancer.

[13] Yokota T, Ando N, Igaki H, Shinoda M, Kato K, Mizusawa J, Katayama H, Nakamura K, Fukuda H, Kitagawa Y (2015). Prognostic Factors in Patients Receiving Neoadjuvant 5-Fluorouracil plus Cisplatin for Advanced Esophageal Cancer (JCOG9907). Oncology.

[14] Chang S, Koo PJ, Kwak JJ, Kim SJ (2016). Changes in Total Lesion Glycolysis Evaluated by Repeated F-18 FDG PET/CT as Prognostic Factor in Locally Advanced Esophageal Cancer Patients Treated with Preoperative Chemoradiotherapy. Oncology.

[15] Gotoh M, Takiuchi H, Kawabe S, Ohta S, Kii T, Kuwakado S, Katsu K (2007). Epidermal growth factor receptor is a possible predictor of sensitivity to chemoradiotherapy in the primary lesion of esophageal squamous cell carcinoma. Jpn J Clin Oncol.

[16] He LR, Liu MZ, Li BK, Rao HL, Deng HX, Guan XY, Zeng YX, Xie D (2009). Overexpression of AIB1 predicts resistance to chemoradiotherapy and poor prognosis in patients with primary esophageal squamous cell carcinoma. Cancer Sci.

[17] He LR, Liu MZ, Li BK, Rao HL, Liao YJ, Zhang LJ, Guan XY, Zeng YX, Xie D (2009). Clusterin as a predictor for chemoradiotherapy sensitivity and patient survival in esophageal squamous cell carcinoma. Cancer Sci.

[18] Kang SY, Han JH, Lee KJ, Choi JH, Park JI, Kim HI, Lee HW, Jang JH, Park JS, Kim HC, Kang S, Oh YT, Chun M (2007). Low expression of Bax predicts poor prognosis in patients with locally advanced esophageal cancer treated with definitive chemoradiotherapy. Clin Cancer Res.

[19] Sohda M, Honjyo H, Hara K, Ozawa D, Suzuki S, Tanaka N, Sano A, Sakai M, Yokobori T, Inose T, Miyazaki T, Ojima H, Higuchi T (2014). L-[3-18F]-alpha-methyltyrosine accumulation as a definitive chemoradiotherapy response predictor in patients with esophageal cancer. Anticancer Res.

[20] Sohda M, Miyazaki T, Honjyo H, Hara K, Ozawa D, Suzuki S, Tanaka N, Sano A, Sakai M, Yokobori T, Nakajima M, Fukuchi M, Kato H (2014). L-[3-(1)(8)F]-alpha-methyltyrosine uptake by lymph node metastasis is a predictor of complete response to CRT in esophageal cancer. Anticancer Res.

[21] Yoon MS, Nam TK, Lee JS, Cho SH, Song JY, Ahn SJ, Chung IJ, Jeong JU, Chung WK, Nah BS (2011). VEGF as a predictor for response to definitive chemoradiotherapy and COX-2 as a prognosticator for survival in esophageal squamous cell carcinoma. J Korean Med Sci.

[22] Zhang JL, Wang HY, Yang Q, Lin SY, Luo GY, Zhang R, Xu GL (2015). Methyl-methanesulfonate sensitivity 19 expression is associated with metastasis and chemoradiotherapy response in esophageal cancer. World J Gastroenterol.

[23] Liang KH, Lin CC, Yeh CT (2011). GALNT14 SNP as a potential predictor of response to combination chemotherapy using 5-FU, mitoxantrone and cisplatin in advanced HCC. Pharmacogenomics.

[24] Yeh CT, Liang KH, Lin CC, Chang ML, Hsu CL, Hung CF (2013). A single nucleotide polymorphism on the GALNT14 gene as an effective predictor of response to chemotherapy in advanced hepatocellular carcinoma. International Journal of Cancer.

[25] Liang KH, Yang PC, Yeh CT (2014). Genotyping the GALNT14 gene by joint analysis of two linked single nucleotide polymorphisms using liver tissues for clinical and geographical comparisons. Oncology Letters.

[26] Lin WR, Hsu CW, Chen YC, Chang ML, Liang KH, Huang YH, Yeh CT (2014). GALNT14 genotype, α-fetoprotein and therapeutic side effects predict post-chemotherapy survival in patients with advanced hepatocellular carcinoma. Mol Clin Oncol.

[27] Liang KH, Lin CL, Chen SF, Chiu CW, Yang PC, Chang ML, Lin CC, Sung KF, Yeh C, Hung CF, Chien RN, Yeh CT (2016). GALNT14 genotype effectively predicts the therapeutic response in unresectable hepatocellular carcinoma treated with transcatheter arterial chemoembolization. Pharmacogenomics.

[28] Wagner KW, Punnoose EA, Januario T, Lawrence DA, Pitti RM, Lancaster K, Lee D, von Goetz M, Yee SF, Totpal K, Huw L, Katta V, Cavet G (2007). Death-receptor O-glycosylation controls tumor-cell sensitivity to the proapoptotic ligand Apo2L/TRAIL. Nat Med.

[29] Eisenhauer EA, Therasse P, Bogaerts J, Schwartz LH, Sargent D, Ford R, Dancey J, Arbuck S, Gwyther S, Mooney M, Rubinstein L, Shankar L, Dodd L (2009). New response evaluation criteria in solid tumours: Revised RECIST guideline (version 1.1). European Journal of Cancer.

[30] Zhang P, Xi M, Li QQ, Hu YH, Guo X, Zhao L, Liu H, Liu SL, Luo LL, Liu Q, Liu MZ (2015). Concurrent cisplatin and 5-fluorouracil versus concurrent cisplatin and docetaxel with radiotherapy for esophageal squamous cell carcinoma: a propensity score-matched analysis. Oncotarget.

[31] Shamseldin HE, Tulbah M, Kurdi W, Nemer M, Alsahan N, Al Mardawi E, Khalifa O, Hashem A, Kurdi A, Babay Z, Bubshait DK, Ibrahim N, Abdulwahab F (2015). Identification of embryonic lethal genes in humans by autozygosity mapping and exome sequencing in consanguineous families. Genome Biol.

[32] Hansen L, Lind-Thomsen A, Joshi HJ, Pedersen NB, Have CT, Kong Y, Wang S, Sparso T, Grarup N, Vester-Christensen MB, Schjoldager K, Freeze HH, Hansen T (2014). A glycogene mutation map for discovery of diseases of glycosylation. Glycobiology.

[33] De Mariano M, Gallesio R, Chierici M, Furlanello C, Conte M, Garaventa A, Croce M, Ferrini S, Paolo Tonini G, Longo L (2015). Identification of GALNT14 as a novel neuroblastoma predisposition gene. Oncotarget.

[34] Li K, Wang J, Ma ZB, Guo GH (2015). Association between clusterin polymorphisms and esophageal squamous cell carcinoma risk in Han Chinese population. Int J Clin Exp Med.

[35] Peng J, Shao N, Peng H, Chen LQ (2013). Prognostic significance of vascular endothelial growth factor expression in esophageal carcinoma: a meta-analysis. J BUON.

[36] Edge SB, Compton CC, Compton CC (2010). The American Joint Committee on Cancer: the 7th edition of the AJCC cancer staging manual and the future of TNM. Ann Surg Oncol.

[37] Oken MM, Creech RH, Tormey DC, Horton J, Davis TE, McFadden ET, Carbone PP (1982). Toxicity and response criteria of the Eastern Cooperative Oncology Group. American Journal of Clinical Oncology.

