# Supplementary material for: GALNT14 genotype as a response predictor for concurrent chemoradiotherapy in advanced esophageal squamous cell carcinoma
Source: Oncotarget. 2017 Mar 16;8(17):29151–60. doi: 10.18632/oncotarget.16253 (PMC5438720; doi:10.18632/oncotarget.16253)
Supplement: Supplementary file 1 [file oncotarget-08-29151-s001.pdf]

GALNT14 genotype as a response predictor for concurrent chemoradiotherapy in advanced esophageal squamous cell carcinoma

Supplementary Materials

SUPPLEMENTARY TABLES

Supplementary Table 1: Distribution of *GALNT14* rs9679162 genotypes in clinical subgroups

	GALNT14 rs9679162 genotype		
	**TT**	**TG**	**GG**
Gender			
Male	27 (25.96%)	48 (46.15%)	29 (27.88%)
Female	1 (25.00%)	3 (75.00%)	0 (0.00%)
Location of cancer			
Upper	6 (28.57%)	11 (52.38%)	4 (19.05%)
Middle	10 (37.04%)	8 (29.63%)	9 (33.33%)
Lower	6 (26.09%)	9 (39.13%)	8 (34.78%)
Cross Two Regions	6 (16.22%)	23 (62.16%)	8 (21.62%)
Metastasis			
Regional lymph node	9 (21.95%)	20 (48.78%)	12 (29.27%)
Distant lymph node	3 (23.08%)	6 (46.15%)	4 (30.77%)
Organ	16 (29.63%)	25 (46.30%)	13 (24.07%)

Supplementary Table 2: Evaluation of the associations between clinical variables with the *GALNT14* genotype "GG" vs. "non-GG" (including TG and TT) using the logistic regression

	***GALNT14* "GG" genotype**		
	**Beta**	**OR, 95% CI**	**P**
Age, years	0.012	1.013, 0.968 – 1.060	0.590
Gender, Male = 1	20.253	>10^8, 0.000 – [a]	0.999
Location of tumor			
Upper = 1	-0.539	0.584, 0.179 – 1.907	0.373
Middle = 1	0.422	1.525, 0.592 – 3.928	0.382
Lower = 1	0.486	1.625, 0.604 – 4.372	0.336
Histology, Poorly differentiated = 1	-0.776	0.460, 0.157 – 1.351	0.158
Tumor stage	-0.184	0.832, 0.441 – 1.567	0.568
Metastasis stage	0.072	1.075, 0.683 – 1.692	0.755
ECOG Stage, greater than one = 1	0.376	1.457, 0.490 – 4.325	0.498
Tumor length, cm	-0.008	0.992, 0.979 – 1.005	0.225
Albumin, g/dL	-0.480	0.619, 0.265 – 1.444	0.267
Alanine transaminase, U/L	0.005	1.005, 0.979 – 1.032	0.695
Creatinine, mg/dL	0.619	1.858, 0.404 – 8.543	0.426
Bilirubin, mg/dL	0.238	1.269, 0.538 – 2.993	0.587
Leukocytes, × 10^9/L	-0.066	0.936, 0.837 – 1.047	0.248
Neutrophil percentage, %	-0.170	0.843, 0.021 – 33.917	0.928
Hemoglobin, g/dL	-0.076	0.927, 0.759 – 1.131	0.455

[a]None of the 5 females were genotype "GG".
